# A Stabilization Device That Promotes the Efficiency of Cardiopulmonary Resuscitation during Ambulance Transportation to the Level as under Non-Moving Conditions

**DOI:** 10.1371/journal.pone.0107960

**Published:** 2014-10-15

**Authors:** Ning-Ping Foo, Jer-Hao Chang, Shih-Bin Su, Kow-Tong Chen, Ching-Fa Cheng, Pei-Chung Chen, Tsung-Yi Lin, How-Ran Guo

**Affiliations:** 1 Department of Emergency Medicine, China Medical University-An Nan Hospital, Taichung, Taiwan; 2 Department of Environmental and Occupational Health College of Medicine, National Cheng Kung University, Tainan, Taiwan; 3 Department of Emergency Medicine, Ditmanson Medical Foundation, Chiayi Christian Hospital, Chia-Yi, Taiwan; 4 Department of Occupational Therapy, College of Medicine, National Cheng Kung University, Tainan, Taiwan; 5 Department of Occupational Medicine, Chi-Mei Medical Center, Tainan, Taiwan; 6 Department of Leisure, Recreation and Tourism Management, Southern Taiwan University of Science and Technology, Tainan, Taiwan; 7 Department of Public Health, College of Medicine, National Cheng Kung University, Tainan, Taiwan; 8 Department of Occupational Medicine, Tainan Municipal Hospital, Tainan, Taiwan; 9 Division of Emergency Medical Services, Tainan City Fire Bureau, Tainan, Taiwan; 10 Department of Mechanical Engineering, Southern Taiwan University of Science and Technology,Tainan, Taiwan; 11 Department of Occupational and Environmental Medicine, National Cheng Kung University Hospital, Tainan, Taiwan; Azienda Ospedaliero-Universitaria Careggi, Italy

## Abstract

**Background:**

The survival rate of patients with out-of-hospital cardiac arrest is low, and measures to improve the quality of cardiopulmonary resuscitation (CPR) during ambulance transportation are desirable. We designed a stabilization device, and in a randomized crossover trial we found performing CPR in a moving ambulance with the device (MD) could achieve better efficiency than that without the device (MND), but the efficiency was lower than that in a non-moving ambulance (NM).

**Purpose:**

To evaluate whether a modified version of the stabilization device, can promote further the quality of CPR during ambulance transportation.

**Methods:**

Participants of the previous study were recruited, and they performed CPR for 10 minutes in a moving ambulance with the modified version of the stabilization device (MVSD). The primary outcomes were effective chest compressions and no-flow fraction recorded by a skill-reporter manikin. The secondary outcomes included back pain, physiological parameters, and the participants' rating about the device after performing CPR.

**Results:**

The overall effective compressions in 10 minutes were 86.4±17.5% for NM, 60.9±14.6% for MND, 69.7±22.4% for MD, and 86.6%±13.2% for MVSD (p<0.001). Whereas changes in back pain severity and physiology parameters were similar under all conditions, MVSD had the lowest no-flow fraction. Differences in effective compressions and the no-flow fraction between MVSD and NM did not reach statistical significance.

**Conclusions:**

The use of the modified device can improve quality of CPR in a moving ambulance to a level similar to that in a non-moving condition without increasing the severity of back pain.

## Introduction

The global average incidence for out-of-hospital cardiac arrest (OHCA) among adults is 55 cases per 100,000 people per year, of which the average survival rates are 9% in Europe and 2% in Asia [1]. In the past few decades, various strategies have been proposed to improve the prognosis of OHCA, but these efforts did not increase the overall survival rate [2]. The survival involves multiple prognostic factors, including the quality of cardiopulmonary resuscitation (CPR) performed in the ambulance during transportation to the hospital.

“High-quality CPR” refers to performing at least 100 chest compressions per minute with a depth of at least 5 cm for each chest compression, allowing enough rebound after each chest compression, and making efforts to avoid any interruption [3]. However, such high-quality CPR is almost impossible to deliver in a moving ambulance [4]–[7]. Performing CPR in a moving ambulance may lead to back pain, wrist pain, and bruises in the forehead from bumping into objects and other people. Rescuers has to cope with frequent abrupt turns, sudden braking, and deceleration and acceleration of the vehicle, and therefore they often have to use one hand for chest compression while using the other to maintain their balance, resulting in poor quality CPR. Some approaches can partially solve these problems (for example, using mechanical CPR devices such as Lucas and AutoPulse), but there are considerable feasibility problems in operating these devices, and economic concerns also limit their use.

In previous studies, we have designed a stabilization device that can improve the quality of CPR in a moving ambulance (Phase I Study) [7]. In a randomized crossover trial, we found that the device could improve the quality of CPR in a moving ambulance, but only 69% of the CPR operations met the requirements for high-quality CPR within 10 minutes. Together with other shortcomings, it could not be formally introduced to clinical practice. Therefore, we modified the design and developed a new device. The current study (Phase II Study) was designed to evaluate whether this new device can promote the quality of CPR in a moving ambulance further.

## Materials and Methods

### Study participants and setting

**Figure 1:**
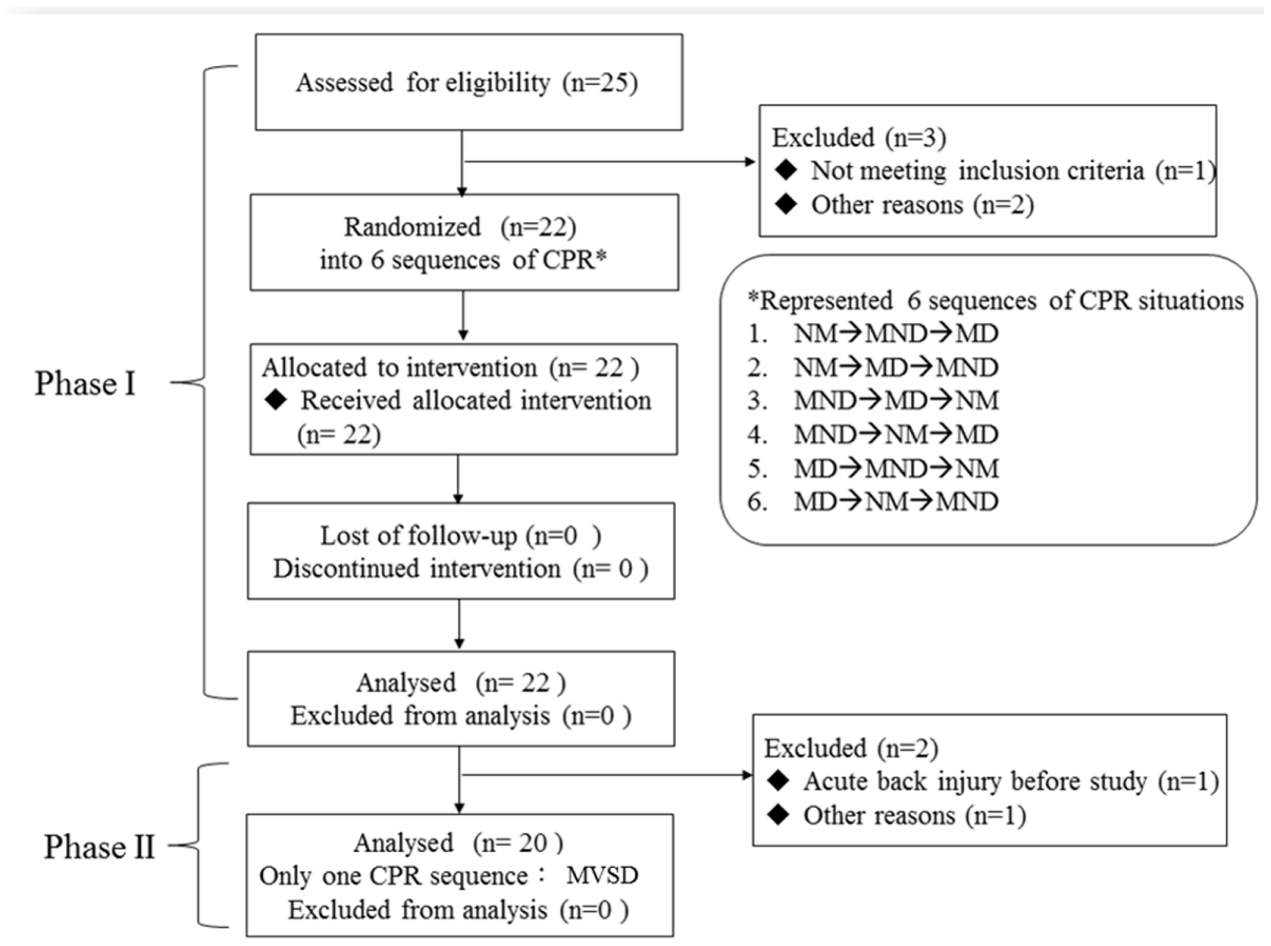
Flowchart of the study. CPR, cardiopulmonary resuscitation; NM: on-moving ambulance; MND: moving ambulance without device; MD: moving ambulance with device; MVSD: Promoted CPR.

In the Phase I Study, 22 ambulance staff from the Tainan City Fire Department in Taiwan participated and performed CPR for 10 minutes each using a Laerdal Resusci-Anne Skill Reporter manikin (Norway) under three conditions, with 72-hour intervals in between: in a non-moving ambulance (NM), in a moving ambulance without the device (MND), and in a moving ambulance with the device (MD). We recruited participants from those who had participated in the Phase I Study and asked them to perform the same 10-minute high-quality [3] CPR session with the new device on the same type of reporter manikin in the same ambulance moving in the same test field (MVSD session). The flowchart of the study was shown in Figure 1.

The participants had at least two years of on-site work experience and had performed CPR in a moving ambulance for a minimum of 20 times. They also had a certificate of basic-life support and a minimum of 8 hours of relevant renewed training courses every year. Candidates were excluded if they had a history of the following: (1) sciatica, (2) intervertebral disc herniation, (3) ankylosing spondylitis, (4) autoimmune disease, (5) spinal surgery, and (6) acute back pain.

The test field was roughly rectangular; each of the two long sides was 250 meters long and straight, while the curving paths on the width were 92 meters and 67 meters long. There were no traffic lights and few other vehicles on the route. The same driver who participated in the Phase I Study was recruited to drive at the same speed: 50±5 km/h along the straight path and 30±5 km/h along the curving path.

As in the Phase I Study, a monitor was set at the right front side of the participant to keep a rate of 100 compressions per minute. The manikin CPR was placed on a stretcher in the ambulance without a hard board underneath.

### Intervention

**Figure 2:**
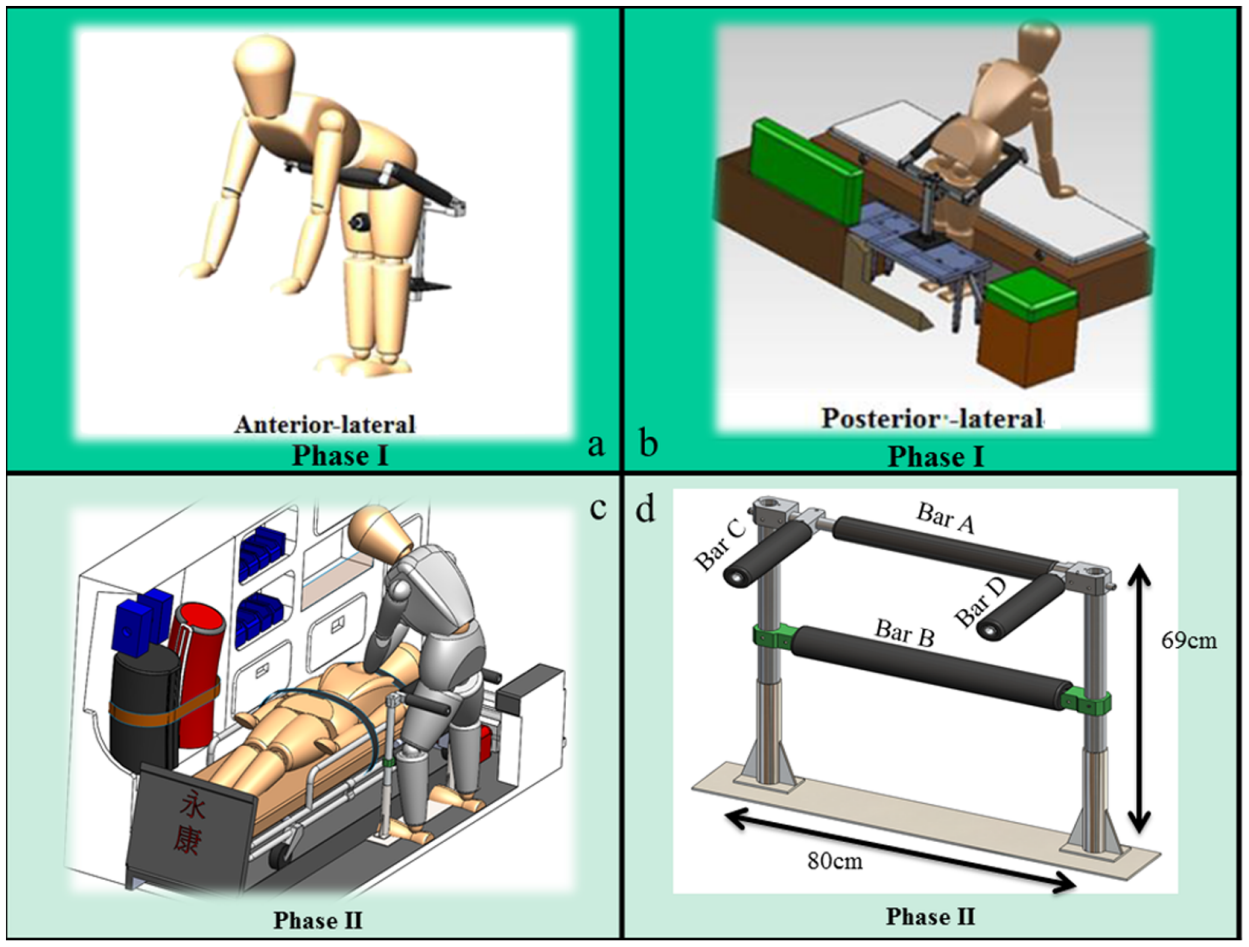
The structures of the stabilization device (Phase I, [a], [b]) and MVSD (Phase II, [c], [d]).

In the Phase I Study, the stabilization device was placed on participant's back and fixed on the floor of the ambulance, with a bar in front of the participant to fix the waist for facilitating CPR. The stabilization device is 28.8 kg in weight and 90 cm in height (Fig. 2).

The new device is 8.9 kg in weight and 69 cm in height. In the Phase II Study, we placed it in front of the participant and set one bar (Bar A, adjustable to fit the participant's height) against the thigh, another (Bar B) against the knees, and one each beside each thigh (Bars C and D) to avoid harsh movements (Fig. 2). A safety belt on each side was also provided to protect the rescuer.

### Outcome assessments

The primary outcomes are indicators of the quality of chest compressions. The skill-reporter manikin recorded the depth, position, and rebound of each chest compression and any interruptions of compressions. With the reported data, we calculated the effective chest compressions (presented as a percentage) and the no-flow fraction, the ratio of the duration without chest compressions to the cardiac arrest time (e.g., a value of 0.25 indicates that the patient did not receive chest compressions for 25% of the cardiac arrest time).

The secondary outcomes were the participants' back pain, physiological parameter, and responses to a questionnaire. We used the Brief Pain Inventory-Short Form (BPI-sf) to assess back pain [8], [9]. The reliability and validity had been evaluated in Taiwanese, and it contained two parts: (1) severity scores, including four subscores for pain at its worst, pain at its least, pain at its average, and pain at the time of answering the questionnaire and (2) interferences score, including seven subscores for general activity, mood, walking ability, work ability, relationships, sleep, and entertainment. For each item, the score ranged from 0 to 10. Participants completed a form within 24 hours before and after test sessions, and we used the changes after the session as the outcomes. Likewise, for physiological parameters we used the differences in blood pressure and heart rate measured with a blood pressure meter before and after the session as the outcomes. The questionnaire included questions concerning the participants' opinions about the new device compared with the old device: whether there was reduced back pain, whether there was increased riding safety for the ambulance technician, whether the device was helpful in improving CPR quality, and whether the respondent would be willing to use the device if it were available on the market. The scores also ranged from 0 to 10, with 5 as no comment, a higher score indicating stronger agreement, and a lower score indicating stronger disagreement.

### Statistical analysis

We reported measurements as “mean ± standard deviation” or “median (inter-quartile range)” and applied the Shapiro-Wilk test to evaluate the normality of the distribution of data. Then, we applied ANOVA for repeat measures and the Bonferroni procedure to evaluate the differences among and between different positions if the data fit the Gaussian distribution, and the Friedman test and Wilcoxon signed-rank test if the data did not fit the Gaussian distribution. All the statistical tests were performed at the two-tailed level of significance at 0.05, and all statistical analyses were performed using SPSS for Windows, Version 17.0 (SPSS Inc., Chicago, U.S.A.).

This study was approved by the Institutional Review Board (IRB) of Ditmanson Medical Foundation, Chiayi Christian Hospital. All the participants gave their written informed consent to participate in this study, and the IRB approved the consent procedure.

## Results

### Participant characteristics

Of the 22 participants of the Phase I Study, one had been relocated to another county and another had suffered an acute back pain episode when he received the invitation (Fig. 1). The 20 participants in the Phase II Study had similar weights (74.6±8.3 *vs.* 73.7±8.2 kg, *p* = 0.229) and body mass index (25.1±2.1 *vs.* 24.7±1.9 kg/m^2^, *p* = 0.199) as those in the Phase I Study, and their heights were 172.4±4.9 cm (ranging between 165 and 180 cm).

### Effective chest compression and no-flow fraction

**Figure 3:**
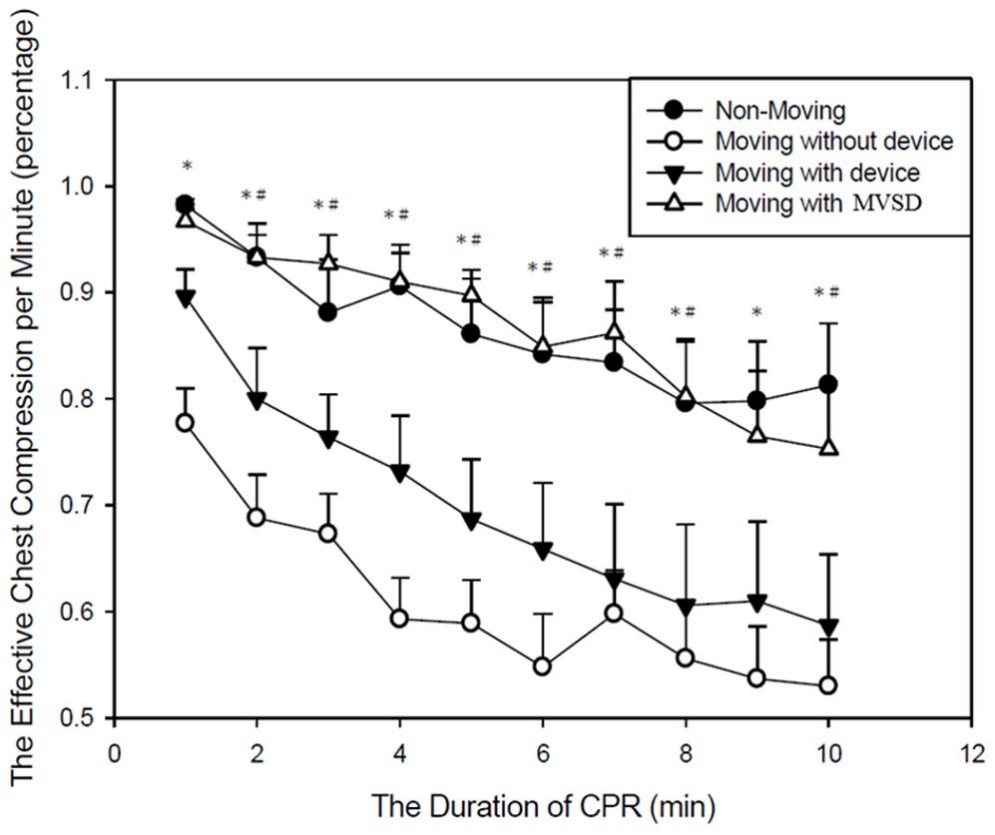
The effective chest compressions among the groups. ANOVA for repeat measurements; Post hoc: Bonferroni test. *Significant difference between MVSD and MND (P<0.05). ^#^Significant difference between MVSD and MD (P<0.05).

**Figure 4:**
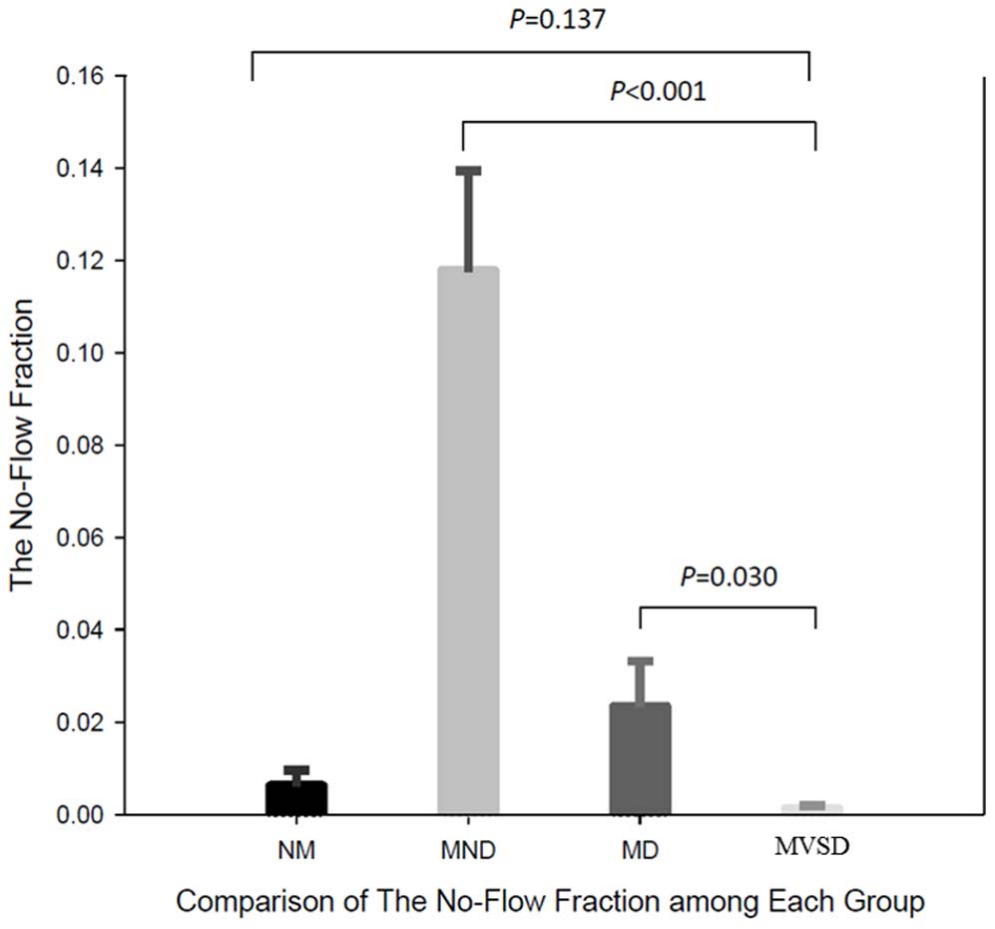
The no-flow fraction among the groups. Paired t test. NM: on-moving ambulance; MND: moving ambulance without device; MD: moving ambulance with device; MVSD: Modified version of stabilization device.

The proportions of effective chest compression during the 10-minute CPR session were 86.4±17.5% for NM, 60.9±14.6% for MND, 69.7±22.4% for MD, and 86.6±13.3% for MVSD (*p*<0.001) (Fig. 3). The no-flow fractions during the session were 0.006±0.016 for NM, 0.118±0.098 for MND, 0.023±0.045 for MD, and 0.001±0.004 for MVSD (*p*<0.001) (Fig. 4). For both outcomes, MVSD was similar to NM, but better than MND and MD (all *p*<0.001).

### Back pain

The changes in pain scores were the BPI-sf scores provided by the participants 24 hours after the CPR operation minus the scores before CPR. The median values of total severity scores were 2.0 (0.0–4.0) for NM, 1.5 (0.0–5.8) for MND, 2.0 (0.0–5.0) for MD, and 2.0 (0.3–8.0) for MVSD (*p* = 0.093) (Table 1). The changes in total interference scores were 0.0 for (0.0–0.0) NM, 0.0 (0.0–1.0) for MND, 0.0 (0.0–0.1) for MD, and 0.5 (0.0–1.8) for MVSD (*p* = 0.033).

**Table 1:** Severity and social interference scores 24 hours after CPR.

	NM	MND	MD	MVSD	*p* value (Friedman test)
	Median (IQR)	Median (IQR)	Median (IQR)	Median (IQR)	
Severity items					
Pain at its worst	0.5 (0.0–2.0)	1.0 (0.0–2.8)	0.5 (0.0–2.8)	0.1 (0.0–2.0)	0.697
Pain at its least	0.0 (0.0–1.0)	0.0 (0.0–1.0)	0.0 (0.0–1.0)	0.0 (0.0–1.8)	0.625
Pain at average	0.0 (0.0–1.0)	0.0 (0.0–1.0)	0.0 (0.0–1.0)	0.5 (0.0–2.0)	0.671
Pain at current	0.0 (0.0–1.0)	0.0 (0.0–1.0)	1.0 (0.0–1.0)	0.5 (0.0–2.0)	0.323
Total pain intensity	2.0 (0.0–4.0)	1.5 (0.0–5.8)	2.0 (0.0–5.0)	2.0 (0.3–8.0)	0.093
Interference items					
General activity	0.0 (0.0–0.0)	0.0 (0.0–0.0)	0.0 (0.0–0.0)	0.0 (0.0–0.0)	0.433
Mood	0.0 (0.0–0.0)	0.0 (0.0–0.0)	0.0 (0.0–0.0)	0.0 (0.0–0.8)	0.053
Walking ability	0.0 (0.0–0.0)	0.0 (0.0–0.0)	0.0 (0.0–0.0)	0.0 (0.0–0.0)	0.572
Normal work	0.0 (0.0–0.0)	0.0 (0.0–0.2)	0.0 (0.0–0.0)	0.0 (0.0–0.0)	0.236
Relations with people	0.0 (0.0–0.0)	0.0 (0.0–0.0)	0.0 (0.0–0.0)	0.0 (0.0–0.0)	0.187
Sleep	0.0 (0.0–0.0)	0.0 (0.0–1.0)	0.0 (0.0–0.0)	0.0 (0.0–0.0)	0.137
Entertainment	0.0 (0.0–0.0)	0.0 (0.0–0.0)	0.0 (0.0–0.0)	0.0 (0.0–0.0)	0.137
Total interference score	0.0 (0.0–0.0)	0.0 (0.0–1.0)	0.0 (0.0–0.1)	0.5 (0.0–1.8)	0.033*

*p<0.05.

### Physiology parameters

**Figure 5:**
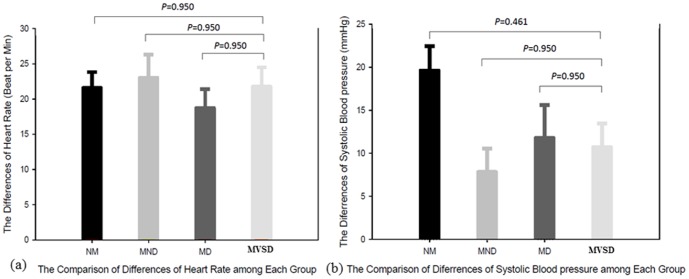
The differences in heart rate (a) and systolic pressure (b) before and after CPR among the group. Paired t test. NM: on-moving ambulance; MND: moving ambulance without device; MD: moving ambulance with device; MVSD: Modified version of stabilization device.

The blood pressure and heart rate of the participants increased after they performed CPR under all four conditions The systolic blood pressures measured before and after the sessions were 135.6±11.1 *vs.* 155.2±10.7 mmHg for NM (*p*<0.001), 139.9±10.1 *vs.* 147.7±10.8 mmHg for MND (*p* = 0.010), 136.8±12.9 *vs.* 148.6±14.7 mmHg for MD (*p* = 0.007), and 132.6±11.5 *vs.* 143.6±14.6 mmHg for MVSD (*p* = 0.001).The heart rates measured before and after the sessions were 76.6±11.2 *vs.* 98.2±13.8 beats/min for NM (*p*<0.001), 79.4±10.3 *vs.* 102.4±15.9 beats/min for MND (*p*<0.001), 77.6±10.3 *vs.* 96.3±20.1 beats/min for MD (*p*<0.001), and 80.1±12.4 *vs.* 101.9±16.5 beats/min for MVSD (*p*<0.001). However, the increases in the rescuers' systolic blood pressure, diastolic pressure, and heart rate were similar among the conditions (Fig. 5).

### Questionnaires

Comparing with the device used in the Phase I Study, participants believed that the new device could more effectively reduce back pain, as evidenced by their back pain scores of 8.0 (7.3–9.0) *vs.* 5.5 (3.0–8.8) (*p* = 0.012). They also believed that the new device could increase the quality of CPR, as evidenced by the scores of 9.0 (8.0–10.0) *vs.* 8.0 (7.0–9.0) (*p* = 0.019). Whereas the scores of riding safety were similar (9.0 [8.3–10.0] *vs.* 9.0 [8.0–9.8], *p* = 0.081), the participants' willingness to use the new device was high, scored 8.0 (7.0–10.0).

## Discussion

High-quality CPR is critical for saving OHCA patients but often not achievable in a moving ambulance. Clinical studies have revealed that the no-flow fraction for CPR in a moving ambulance was 0.27–0.57 [5], [6], [10], [11]. As to effective chest compression, studies using reporter manikins presented different results. It was only 21% for emergency care provided to patients on a moving stretcher [12], but reached 45.6%–95% in a moving ambulance [4], [7], [13]. The quality of CPR can be affected by multiple factors such as ambulance speed, acceleration force, and sudden braking force. Research has shown that in an ambulance moving at 30 km/hour, the no-flow fraction could be 0.289, which increased as the vehicle's speed increased [14]. In a moving ambulance, if strong acceleration accounted for 60% of the total transport time, the no-flow fraction could reach 0.42 [15]. The current study showed that the new device could promote the quality of CPR performed in a moving ambulance, as indicated by the no-flow fraction (0.001±0.004) and effective chest compressions (an average of 92.7±0.1% in the first 5 minutes and 86.6±13.3% in 10 minutes). In addition, no physical burden or more serious back pain related to the provision of high-quality CPR was reported by the participants.

It is challenging to design a stabilization device with minimal shortcomings. In the Phase I Study, the device proved able to improve the quality of CPR. However, as discussed in the Introduction, it has various drawbacks, including a relatively low effective chest compression rate (77.5±0.2% in 5 minutes and 69.7±22.4% in 10 minutes). Compared with MND, that device could improve the CPR quality in the first 5 minutes, but the improvement could not last for 10 minutes. Even in the first 5 minutes, the quality was poorer than that in NM. In addition, although the device was effective, ambulance staffs were not willing to use it because it was very heavy and not foldable, and with the device in the rear of the ambulance, people could not pass easily. The space in an ambulance is extremely valuable, and the device became an obstacle in the ambulance, especially when it is not on OHCA duty. Furthermore, the device uses a bar to fix the rescuer's waist and abdomen during CPR that is not suitable for people who are too tall or short. For a tall person, the bar does not provide effective fixing, and for a short person, it makes CPR more difficult because it reduced the downward force.

After two years of experiments, we developed a new device to solve the above problems. It is placed in front of the rescuer and thus can reduce the impact on accessibility. Its small size and weight enable it to be placed in the ambulance without blocking any operations. In addition, from the current study, we found it unnecessary to adjust the height of Bar A because the location at which Bar A is fixed on the thigh to be helpful had a wide range. The thigh itself is relatively long, so the bar's location had a minor effect on the force that the participant can provide. Moreover, the current study also confirmed that the resulting quality of the new device is comparable to that of CPR performed in a non-moving vehicle.

An ideal mechanical assistance device should have a satisfactory performance comparable that of manually performed CPR. In theory, the use of a mechanical assistance device such as Lucas or AutoPulse poses no rescuer fatigue problems, and the no-flow fraction can be zero, even in a moving ambulance. A study using Lucas on a reporter manikin in a moving vehicle found the effective chest compression rate could reach 99.96% [16]. However, another study argued that this device was actually not very beneficial and could exert negative impacts on the neurological prognosis of OHCA patients [17], and a randomized trial found no differences in the 4-hour survival rate of OHCA patients between the applications of a mechanical device and traditional manual CPR [18]. In fact, some comprehensive reviews failed to find any definitive evidence to support the use of mechanical assistance devices [19], [20]. Moreover, from the economic perspective, it is not practical to promote mechanical devices in all places. For example, the high price of AutoPulse makes it impossible to extensively place it ambulances in most countries, even in the United States and European countries. In contrast, it cost us less than 1/40 the price of an AutoPulse to make the new device. Another consideration is the transportation time. In urban areas, it is usually not long, and the new device can be very effective. In suburban areas, however, it can easily go beyond 10 minutes and may be hours. In those cases, rescuer fatigue makes it necessary to install mechanical devices.

Back pain is a difficult problem for ambulance staff to avoid [21]. We attempted to design a device that would reduce back pain, but neither the Phase I nor the Phase II Study achieved this goal. We believe the repeated action of pushing down using the strength of the waist and back can easily cause back pain. Interestingly, while the new device did not decrease back pain scores in our assessment with the BPI-sf, in the questionnaire survey, participants believed that the new device could reduce the severity of back pain. This might be due to the difference in the time of survey. The formal survey was completed up to 24 hours after CPR was performed, while the latter was completed immediately after the CPR. Whereas the total interference score with the use of the new device was higher than those in the other three conditions, it was relatively low—no more than 1.8 with an average of 0.5 out of a scale from 0 to 10, which was not clinically significant.

With the use of the new device, the increases in participants' systolic and diastolic blood pressures and heart rates did not reach statistical significance. This suggested that performing CPR for 10 minutes did not cause remarkable additional physiological burden compared with the other three conditions.

This study compared the participants to themselves in the same environment, including the ambulance, path, driver, manikin, and the speed, to minimize interference with the results. A major potential limitation was the two-year interval between the Phase I and Phase II. We believe this would not cause a remarkable impact based on the following three main considerations. First, in the Phase I Study, the participants were not aware of the plan of the Phase II Study and thus would not perform any additional practices. Second, in humans, physical ability peaks at the age of 25 to 30 years, and afterwards physical strength and muscle strength begin to gradually decline. The average age of our participants was 32.4±5.5 in Phase I and 34.4±5.5 in Phase II, and so their physical ability should have been declining, not improving. Third, we performed a further analysis by subgrouping the participants in the Phase I Study into two age groups, one ≤32 years and the other group>32 years. The effective chest compression rate in the first 10 minutes under NM conditions was lower in the older group (91.0±0.1% *vs.* 81.0±0.2%), but the difference did not reach statistical significance (*p* = 0.704).

## Conclusions

Our study showed that for performing CPR in a moving ambulance, the use of the new device could increase effective chest compressions and reduce the no-flow fraction to levels similar to those in the non-moving condition, with no extra physiological burden or negative impacts on the severity of rescuers' back pain.

## References

[1] Berdowski J, Berg RA, Tijssen JG, Koster RW (2010) Global incidences of out-of-hospital cardiac arrest and survival rates: Systematic review of 67 prospective studies. Resuscitation 81: 1479–1487 10.1016/j.resuscitation.2010.08.006 20828914

[2] Sasson C, Rogers MA, Dahl J, Kellermann AL (2010) Predictors of survival from out-of-hospital cardiac arrest: a systematic review and meta-analysis. Circ Cardiovasc Qual Outcomes 3: 63–81 10.1161/CIRCOUTCOMES.109.889576 20123673

[3] Travers AH, Rea TD, Bobrow BJ, Edelson DP, Berg RA, et al (2010) Part 4: CPR overview: 2010 American Heart Association Guidelines for Cardiopulmonary Resuscitation and Emergency Cardiovascular Care. Circulation 2: S676–684 10.1161/CIRCULATIONAHA.110.970913 20956220

[4] Havel C, Schreiber W, Riedmuller E, Haugk M, Richling N, et al (2007) Quality of closed chest compression in ambulance vehicles, flying helicopters and at the scene. Resuscitation 73: 264–270. 17276575 10.1016/j.resuscitation.2006.09.007

[5] Ødegaard S, Olasveengen T, Steen PA, Kramer-Johansen J (2009) The effect of transport on quality of cardiopulmonary resuscitation in out-of-hospital cardiac arrest. Resuscitation 80: 843–848 10.1016/j.resuscitation.2009.03.032 19477573

[6] Olasveengen TM, Wik L, Steen PA (2008) Quality of cardiopulmonary resuscitation before and during transport in out-of-hospital cardiac arrest. Resuscitation 76: 185–190. 17728039 10.1016/j.resuscitation.2007.07.001

[7] Foo NP, Chang JH, Su SB, Lin HJ, Chen KT, et al (2013) A stabilization device to improve the quality of cardiopulmonary resuscitation during ambulance transportation: A randomized crossover trial. Resuscitation 84: 1579–1584 10.1016/j.resuscitation.2013.06.015 23816898

[8] Cleeland CS (1985) Measurement and prevalence of pain in cancer. Semin Oncol Nurs 1: 87–92. 3849841 10.1016/s0749-2081(85)80041-3

[9] Ger LP, Ho ST, Sun WZ, Wang MS, Cleeland CS (1999) Validation of the Brief Pain Inventory in a Taiwanese population. J Pain Symptom Manage 18: 316–322. 10584454 10.1016/s0885-3924(99)00087-1

[10] Valenzuela TD, Kern KB, Clark LL, Berg RA, Berg MD, et al (2005) Interruptions of chest compressions during emergency medical systems resuscitation. Circulation 112: 1259–1265. 16116053 10.1161/CIRCULATIONAHA.105.537282

[11] Wik L, Kramer-Johansen J, Myklebust H, Sørebø H, Svensson L, et al (2005) Quality of cardiopulmonary resuscitation during out-of-hospital cardiac arrest. JAMA 293: 299–304. 15657322 10.1001/jama.293.3.299

[12] Kim JA, Vogel D, Guimond G, Hostler D, Wang HE, et al (2006) A randomized, controlled comparison of cardiopulmonary resuscitation performed on the floor and on a moving ambulance stretcher. Prehosp Emerg Care 10: 68–70. 16418093 10.1080/10903120500373108

[13] Stone CK, Thomas SH (1995) Can correct closed-chest compressions be performed during prehospital transport? Prehosp Disaster Med10: 121–123. 10.1017/s1049023x00041856 10155415

[14] Chung TN, Kim SW, Cho YS, Chung SP, Park I, et al (2010) Effect of vehicle speed on the quality of closed-chest compression during ambulance transport. Resuscitation 81: 841–847 10.1016/j.resuscitation.2010.02.024 20378237

[15] Kurz MC, Dante SA, Puckett BJ (2012) Estimating the impact of off-balancing forces upon cardiopulmonary resuscitation during ambulance transport. Resuscitation 83: 1085–1089 10.1016/j.resuscitation.2012.01.033. 22306258

[16] Fox J, Fiechter R, Gerstl P, Url A, Wagner H, et al (2013) Mechanical versus manual chest compression CPR under ground ambulance transport conditions. Acute Card Care 15: 1–6 10.3109/17482941.2012.735675 23425006

[17] Hallstrom A, Rea TD, Sayre MR, Christenson J, Anton AR, et al (2006) Manual Chest compression vs use of an automated chest compression device during resuscitation following out-of-hospital cardiac arrest: a randomized trial. JAMA 14;295: 2620–2628. 10.1001/jama.295.22.2620 16772625

[18] Rubertsson S, Lindgren E, Smekal D, Östlund O, Silfverstolpe J, et al (2014) Mechanical chest compressions and simultaneous defibrillation vs conventional cardiopulmonary resuscitation in out-of-hospital cardiac arrest: the LINC randomized trial. JAMA 311: 53–61 10.1001/jama.2013.282538 24240611

[19] Ong ME, Mackey KE, Zhang ZC, Tanaka H, Ma MH, et al (2012) Mechanical CPR devices compared to manual CPR during out-of-hospital cardiac arrest and ambulance transport: a systematic review. Scand J Trauma Resusc Emerg Med 20: 39 10.1186/1757-7241-20-39 22709917 PMC3416709

[20] Brooks SC, Hassan N, Bigham BL, Morrison LJ (2014) Mechanical versus manual chest compressions for cardiac arrest. Cochrane Database Syst Rev 2: CD007260 10.1002/14651858.CD007260.pub3 24574099

[21] Jones AY (2004) Can cardiopulmonary resuscitation injury the back? Resuscitation 61: 63–67. 15081183 10.1016/j.resuscitation.2003.12.007

